# Tailoring Biosecurity Advice in Dairy Cattle Farms: Decision Analysis to Estimate the Most Cost‐Effective Option

**DOI:** 10.1155/tbed/1472113

**Published:** 2026-08-31

**Authors:** Fernando Duarte, Alberto Allepuz, Arnau Àlvarez, Jordi Casal, Natalia Ciria, Bodil Højlund, Jehan Ettema, Giovanna Ciaravino

**Affiliations:** ^1^ Departament de Sanitat i Anatomia Animals, Universitat Autònoma de Barcelona (UAB), Bellaterra, Barcelona 08193, Spain, uab.cat; ^2^ Departament de Medicina i Cirurgia Animals, Universitat Autònoma de Barcelona (UAB), Bellaterra, Barcelona 08193, Spain, uab.cat; ^3^ SimHerd A/S, Agro Business Park, Niels Pedersens Allé 2, Tjele 8830, Denmark

**Keywords:** bovine viral diarrhea (BVD), calculator tool, decision tree, economic analysis, herd simulation, risk analysis

## Abstract

The implementation of biosecurity measures on dairy farms is influenced by multiple factors, including farmers’ experience, perception of disease risk, social influences, and epidemiological and economic factors. The aim of this study was to identify the biosecurity option providing the highest economic return for dairy farms. To achieve this, a biosecurity cost calculator was developed using a database of prices associated with the different components required to implement each biosecurity measure. The probability of pathogen introduction was estimated through a quantitative risk assessment model, while the economic consequences of a disease outbreak were evaluated using the stochastic dynamic model SimHerd. The outputs from these tools were combined through decision tree analysis, integrating scenario costs with the estimated probability of occurrence. For each scenario, the expected monetary value (EMV) was calculated, and the alternative with the highest EMV was considered the most profitable option. Bovine viral diarrhea (BVD) was used as a case study because of its well‐documented effects on dairy production. The framework was applied to three dairy farms located in northeastern Spain, where biosecurity and production information were collected. The annual biosecurity cost per cow was estimated at €27.49, €44.50, and €71.94 for farms A, B, and C, respectively. The annual probability of BVD introduction under current conditions was 2.66%, 15.70%, and 0.36% for the same farms. Simulation of a BVD outbreak indicated an average reduction in gross margin (GM) of ~6% during the first 5 years after introduction. Across the evaluated scenarios, the provision of dedicated boots for cattle transport drivers was identified as the most economically advantageous measure. The proposed framework enabled the comparison of biosecurity interventions by jointly considering implementation costs and risk reduction. By demonstrating the economic and epidemiological value of biosecurity, this approach may support decision‐making and encourage wider adoption of preventive measures on dairy farms.

## 1. Introduction

The introduction of infectious agents into dairy herds may compromise animal health and welfare and can also generate considerable economic losses. Several diseases affecting cattle production, including bovine viral diarrhea (BVD), paratuberculosis, neosporosis, and infectious bovine rhinotracheitis, have been associated with reduced farm profitability [1–4]. The application of biosecurity measures, intended to prevent or reduce pathogen transmission [5], is widely recognized as an essential component of disease prevention programmes. Effective biosecurity can reduce the likelihood of disease outbreaks, improve herd health, and contribute to lowering the use of antimicrobial treatments [6, 7].

Despite these advantages, the adoption and maintenance of biosecurity measures remain challenging in many livestock systems. Decisions regarding the adoption and implementation of biosecurity measures are influenced by a combination of factors, including farmers’ previous experiences, their perception of disease risks, social influences, and local epidemiological conditions [8, 9]. Economic considerations also play an important role. Because farm resources are finite, investments in biosecurity must compete with other production priorities, and implementation costs are frequently perceived as a barrier [10–12]. Nevertheless, farmers may be more willing to adopt biosecurity measures when their expected economic benefits are clearly demonstrated [13, 14]. For this reason, economic evaluations can support both decision‐making and communication strategies aimed at improving the uptake of biosecurity practices [8, 10, 12, 15].

Although the economic implications of biosecurity have received increasing attention, the evidence remains limited, particularly for dairy cattle production. Previous studies have estimated the costs and benefits of selected biosecurity measures in different livestock sectors, including swine, cattle, and poultry production systems [16–19]. These investigations have contributed valuable information regarding the economic value of biosecurity interventions. However, most studies have evaluated a restricted number of measures or have focused on production systems other than dairy farms.

Selecting the most appropriate biosecurity strategy requires consideration not only of implementation costs but also of the probability of pathogen introduction and the economic consequences associated with disease occurrence [20, 21]. Therefore, the objective of the present study was to identify the most cost‐effective biosecurity strategy for dairy cattle farms through an integrated framework combining three key components: the cost of implementing biosecurity measures, the probability of pathogen introduction, and the economic impact of disease introduction. The proposed approach was designed to support farm‐level decision‐making by providing veterinarians and advisors with quantitative information to compare alternative biosecurity options. While the framework is illustrated here using dairy cattle farms and BVD as a case study, it is intended as a proof of concept and may be adapted to other diseases and production settings through the use of disease‐specific epidemiological and economic parameters. As part of this framework, a biosecurity cost calculator was developed to estimate the costs associated with implementing different biosecurity measures. The methodology and its application are presented in detail in this study.

## 2. Materials and Methods

A decision‐analysis methodology was used to determine the most cost‐effective biosecurity strategy at the farm level. The analysis combined three sources of information: (i) the estimated cost of implementing biosecurity measures, (ii) the economic consequences of disease introduction, and (iii) the probability of pathogen introduction.

To integrate these components, a biosecurity cost calculator was developed and combined with outputs generated by two previously existing models, SimHerd and FarmRisk. The resulting framework was implemented through decision tree analysis, allowing comparison between the current farm situation and alternative biosecurity scenarios from an economic perspective.

BVD was selected as the model disease to demonstrate the application of the framework. Data from three dairy cattle farms located in Catalonia (Spain) were used for this purpose.

### 2.1. Estimation of Biosecurity Cost

Biosecurity costs were estimated using a deterministic calculator developed in Microsoft Excel (available upon request).

The calculator was organized according to four main pathways through which pathogens may enter a farm: (i) animal introduction, (ii) visitors and vehicle access, (iii) feed and water management, and (iv) pest control.

A set of biosecurity measures was defined for each pathway. Measures related to animal introduction included quarantine procedures, quarantine facilities, exclusive transport of incoming animals, and diagnostic testing. Measures associated with visitors and vehicles included perimeter fencing, visitor and vehicle control, mortality management, and worker hygiene practices. Feed and water management considered feed storage systems and drinking water treatment, whereas pest control measures addressed rodents and both flying and crawling insects.

Using these four routes, an inventory of specific biosecurity measures typically employed on dairy farms was compiled. A summary of the biosecurity measures included in each route is presented in Table 1.

**Table 1:** Biosecurity measures by pathway of introduction included in the cost calculator.

Pathway of introduction	Biosecurity measure	Number of farm‐level inputs^a^
Animal introduction	Quarantine	16
	Transport of incoming animals	13
	Diagnostic testing for incoming animals	8
Farm access by visitors and vehicles	Farm perimeter fencing	19
	Visitor control	6
	Transport of outgoing animals	27
	Mortality management	3
	Worker hygiene protocols	3
Feed storage and water treatment	Feed storage	7
	Drinking water treatment	2
Pest control	Rodent control	4
	Flying and crawling insect control	9

^a^Farm‐level inputs refer to the number of parameters required for the cost estimation of the corresponding biosecurity measure gathered through the farm questionnaire.

**Figure 1:**
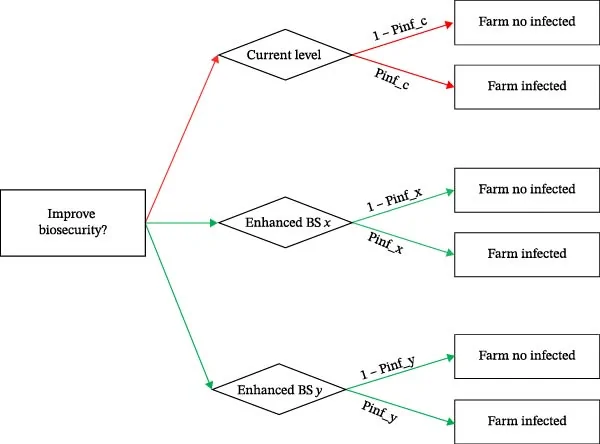
Decision tree used for the economic evaluation of biosecurity strategies in dairy cattle farms. Three alternative biosecurity scenarios were assessed: the existing farm biosecurity level (“Current level”) and two improved biosecurity options (“Enhanced BS x” and “Enhanced BS y”). For each scenario, the probability of BVD introduction depends on the biosecurity measures in place. Under the current situation, the probability of introduction is represented by Pinf_c and the probability of no introduction by (1 − Pinf_c). The same structure applies to the enhanced biosecurity scenarios, represented by Pinf_x and Pinf_y.

To estimate the cost of applying each listed biosecurity measure, the calculator used inputs from three main data sources: (1) a price database, (2) fixed parameters, and (3) data collected from farms. The first two sources were predefined within the tool, meaning users were not required to modify or input this information. To operate the calculator, users simply needed to provide the relevant information about their farm through a standardized questionnaire included in the calculator tool. The full survey is available in Supporting Information Annex 1, Figure 1.S1.

#### 2.1.1. Price Database

Each biosecurity measure was divided into its individual cost components (e.g., materials, equipment, and labor). This process resulted in 75 cost items that formed the basis of the price database (Supporting Information Annex 1, Table 1.S1).

Several cost items could be associated with the same biosecurity measure, allowing users to select the alternative that best matched their farm conditions. For each item, between one and three prices were collected from publicly available sources, mainly supplier websites. Only suppliers operating in Spain or distributing products within the country were included in the database.

Price information was collected during 2024 and expressed in euros, including value‐added tax (VAT; 21%). When more than one price was available for a given item, the mean value was used as the final unit cost estimate. No extreme values were identified among the collected observations. The source of every price included in the database was recorded and documented.

#### 2.1.2. Fixed Parameters

The fixed parameters used for cost estimation for each farm, considering their respective herd sizes and characteristics, were derived from scientific literature, good‐practice guidelines, and consultations with service providers. These parameters included the time required by veterinarians and farm personnel to collect samples from incoming animals, the time needed to change clothing and footwear, the time allocated to pest‐trap inspections, water consumption per animal, the amount and dosage of disinfectants used, and animal transport costs. The implementation time associated with each biosecurity measure was established through consultation with experienced veterinarians and was applied uniformly across all farms.

#### 2.1.3. Farm‐Specific Data

A questionnaire was incorporated into one of the Excel worksheets (Supporting Information Annex 1, Figure 1.S1) to collect both general farm information and data related to the four pathogen‐introduction pathways described previously. The questionnaire contained the information required to estimate the cost of the different biosecurity measures. General farm variables included the herd size, number of workers, working hours, and wages, allowing the cost calculations to be adapted to the characteristics of each farm. The number of farm‐level inputs required for each pathway is presented in Table 1, whereas the complete list of collected variables is provided in Supporting Information Annex 1, Table 2.S2.

#### 2.1.4. Integration of Data

Inputs obtained from the three data sources (i.e., price database, fixed parameters, and farm‐specific information) were combined within a dedicated Excel worksheet. Biosecurity costs were estimated both as total investment costs and as annualized costs that accounted for the useful life of the different components. Annualization was performed using straight‐line depreciation without applying a discount rate. This procedure was applied to infrastructure and equipment included in the calculator, such as perimeter fencing, access control systems, and feed storage facilities.

Using this approach, the calculator estimated the total biosecurity investment on the farm, the annualized biosecurity cost, and the annualized cost per cow based on the number of lactating and dry cows. Outputs were reported both as overall costs and broken down according to the different pathways of pathogen introduction.

The calculator also enabled the evaluation of additional biosecurity measures not currently being implemented on the farm. These measures were incorporated into alternative scenarios, for which both total and annualized costs were estimated. Updated summaries were then generated, presenting biosecurity costs for each scenario in both aggregated form and by pathway of introduction.

### 2.2. Estimating the Cost of a Disease Introduction

The economic consequences of disease introduction (hereafter referred to as the outbreak cost) were estimated using SimHerd [22]. SimHerd is a mechanistic, stochastic, and dynamic simulation model representing a dairy herd and its replacement animals. The model has previously been applied in several studies evaluating the economic effects of management changes and production‐related diseases in dairy cattle [22–24].

In brief, SimHerd models individual animals over time using variables such as age, parity, lactation stage, milk production, reproductive performance, and health status. Herd dynamics were simulated on a weekly basis for both cows and heifers.

The effects of BVD introduction on health and production performance were obtained from the published literature. Parameters included in the analysis were an increased risk of mastitis, a reduced conception rate, and decreased milk yield. The proportional changes applied in the simulations are shown in Table 2, whereas baseline herd production and health parameters are provided in Supporting Information Annex 1 (Table S1.3).

**Table 2:** Proportional changes in cow‐level parameters due to BVD introduction used for herd‐level cost estimation.

Parameter	Proportional change (%)	Reference
Mastitis risk	7.1% (+)	[25]
Conception rate	26.0% (−)^a^	[18, 26]
Milk yield	2.1% (−)^a^	[27, 28]

^a^Mean value of the parameter change reported in literature.

The parameter changes were applied to the production data of the farms included in the study using SimHerd. To avoid double counting of losses, the effects of increased mastitis risk and reduced conception rate were simulated separately in order to estimate their individual contributions to changes in the milk yield. The results showed that part of the observed reduction in milk production was already explained by the 2.1% decrease reported in Table 2. Therefore, the assumed reduction in milk yield was adjusted to account for the effects mediated through mastitis and the conception rate. Based on these results, the contribution of each parameter was adjusted to obtain the reported mean parameter changes.

The main economic output generated by SimHerd was gross margin (GM), defined as the difference between farm revenues and variable production costs. Revenues included income from milk sales, slaughtered cows, and livestock sales, whereas variable costs included feed, breeding expenses, veterinary costs, and other production‐related expenditures such as bedding:

(1) Gross margin GM=total revenue −variable costs.

Model outputs were obtained from 10‐year herd simulations. However, the economic impact of BVD introduction was estimated using the average GM observed during the first 5 years after disease introduction. This period was selected to capture both the immediate production losses and the subsequent herd‐level adjustments following the introduction of the disease. The average annual GM of the infected scenario was therefore compared with the corresponding average annual GM obtained under the BVD‐free scenario.

For the purposes of this study, the outbreak cost was defined as the difference between the GM under baseline conditions and the GM obtained after modifying production parameters to reflect the effects of BVD introduction (Equation (2). All economic outputs were expressed in euros.

(2) Cost_outbreak = GM_noBVD − GM_BVD,

where

• GM_noBVD: average GM during the first 5 years of simulation under baseline (BVD‐free) conditions.
• GM_BVD: average GM during the first 5 years of simulation following BVD introduction.

### 2.3. Estimating the Probability of BVD Introduction

The annual probability of BVD introduction was estimated for each farm using a quantitative stochastic risk assessment model (farmrisk.eu) [29, 30]. Introduction events were modeled through a series of conditional probabilities across independent pathways, and uncertainty was incorporated using Monte Carlo simulation (1000 iterations).

The animal movement pathway considered purchases, external rearing, pasture movements, and livestock markets. For this pathway, the probability that introduced animals were already infected at origin (based on herd‐level and within‐herd prevalence in the region of origin), became infected through contact with animals from other herds, or remained undetected after premovement or quarantine testing was estimated. The probability of infection during transport was also included. For visits involving people and vehicles, the model estimated the probability that visitors had recently contacted infected cattle, that fomites were contaminated, and that on‐farm biosecurity measures (e.g., dedicated equipment and cleaning practices) reduced the risk of introduction, taking into account the visit frequency. The neighboring‐farm pathway included both direct fence‐line contact and indirect contacts associated with local farm density and disease prevalence. Pathway‐specific annual probabilities were subsequently combined to obtain the overall annual probability of introduction.

Quantitative information collected through the farm questionnaire (e.g., visit frequency, quarantine duration, and number of neighboring farms) was directly incorporated into the calculations. Qualitative responses (e.g., yes/no or always/sometimes/never) were converted into probability values to parameterize events such as quarantine implementation, vehicle sharing, and the use of dedicated boots or equipment by visitors. Some variables were also used to link farm‐specific information with pathogen‐ and region‐specific parameters, such as assigning BVD prevalence according to the reported origin of purchased animals. When questionnaire responses did not provide precise values (e.g., “don’t know”), the corresponding inputs were treated as uncertain and represented using predefined probability distributions based on published data describing common regional practices and, when necessary, expert opinion.

BVD‐specific inputs, including prevalence, diagnostic test sensitivity, transmission probabilities, and environmental survival, were derived from published sources and regional surveillance data and were modeled as probability distributions to account for uncertainty.

#### 2.3.1. Scenario Definition

The risk assessment model was also used to evaluate the effect of implementing additional biosecurity measures on the probability of BVD introduction.

A total of 10 predefined external biosecurity measures were assessed individually. These measures covered areas such as the quarantine of incoming animals, diagnostic testing, animal transport, cleaning and disinfection procedures, use of farm equipment, provision of farm‐specific clothing and footwear, and direct contact with farm animals. Based on the simulation results, the measures were ranked according to their ability to reduce the probability of introduction. The three measures showing the greatest risk reduction potential were subsequently selected for evaluation of their economic cost.

### 2.4. Estimating the Cost‐Effectiveness of Farm Biosecurity

The economic evaluation was performed using decision tree analysis. This methodology was selected because it incorporates alternative scenarios together with their associated costs and probabilities of occurrence [12, 31]. The expected monetary value (EMV) was calculated for each branch of the decision tree and used to compare the different biosecurity options. In the present study, EMV was expressed as an expected economic outcome, where negative values represent costs. Consequently, the preferred strategy was the one associated with the highest EMV, corresponding to the lowest expected economic loss.

The EMV of each scenario is calculated as follows:

(3) EMVi=Pinfi×Cost_EBSi+Cost_outbreak+1−Pinfi×Cost_EBSi,

where

• “*i*”: a subscript indicating each scenario (i.e., the implementation of each selected biosecurity measure).
• Pinf_(*i*)_: probability of BVD introduction under each scenario ‘*i*’.
• Cost_EBS_(*i*)_: biosecurity cost associated with the implementation of the selected measure.
• Cost_outbreak: farm‐level cost of BVD introduction. This value was assumed to remain constant across the different biosecurity scenarios (see Equation (2)).

Each branch of the decision tree (Figure 1) represented a specific probability of BVD introduction under the corresponding biosecurity scenario.

### 2.5. Sensitivity Analysis

A sensitivity analysis was performed to evaluate the influence of three key input parameters on the decision‐analysis model: the probability of BVD introduction, the cost associated with BVD introduction, and the cost of biosecurity implementation. The analysis was based on data from Farm A, described in the following section.

For the baseline branch of the decision tree ("current situation"), the probability of BVD introduction was gradually increased up to 50%. The proportional reduction associated with each additional biosecurity measure was adjusted accordingly. This procedure allowed the effect of biosecurity measures to be assessed across a range of predefined probabilities of BVD introduction. For example, assuming a 10% probability of BVD introduction under the current situation of Farm A and a relative reduction of 41.6%, implementation of the measure “Provide boots to all drivers” reduced the probability of introduction to 5.8%. Additional details regarding the proportional reductions applied are provided in Supporting Information, Annex 3 (Table 4.S3).

The estimated cost of BVD introduction was then increased in increments of 10% up to twice the original value. Likewise, the cost of implementing biosecurity measures was increased by 10% intervals up to a maximum of two times the initial estimate. Following each parameter adjustment, the difference in EMVs between the current situation and the alternative biosecurity scenarios was calculated to evaluate the sensitivity of the model outputs to changes in the input parameters.

### 2.6. Data Collection

Dairy farms were recruited through the professional network of one of the authors and participated voluntarily in the study. As a selection criterion, farms were required to be free of BVD to facilitate the subsequent analyses. Three dairy cattle farms located in Catalonia, Spain (northeastern Iberian Peninsula), were visited between October and November 2024.

During the farm visits, information covering the previous 12 months was collected, including production and economic records as well as details of the biosecurity measures implemented on each farm. These data were used as inputs for the risk assessment model, the biosecurity cost calculator, and the decision‐analysis framework. A summary of the characteristics of the participating farms is presented in Table 3.

**Table 3:** General description of the three visited dairy farms in Catalonia between October and November 2024.

Variable	Farm A	Farm B	Farm C
Number of cows	724	247	79
Energy‐corrected milk (ECM, kg per cow‐year)	13,141	13,602	9567
Number of calvings	646	214	60
Cow mortality	5%	11%	9%
Mastitis incidence	60%	20%	26%
Conception rate—cows	36%	49%	31%
Conception rate—heifers	41%	43%	24%

## 3. Results

### 3.1. Farm Biosecurity Cost

The annualized biosecurity cost per cow was estimated at €27.49, €44.50, and €71.94 for farms A, B, and C, respectively. Pest control represented the largest annual biosecurity expenditure on farms A and C. In contrast, animal introduction was the most expensive biosecurity category on farm B, mainly due to the presence of a quarantine system.

Differences between total and annualized biosecurity costs ranged from 56% to 87%. These differences were largely associated with measures requiring substantial initial investments, including farm fencing, access control systems, and feed storage infrastructure, which generally have useful lifespans of 10–20 years. A detailed breakdown of biosecurity costs is presented in Table 4.

**Table 4:** Cost of implemented biosecurity in the three dairy farms studied.

Cost category	Farm A	Farm B	Farm C
Current biosecurity cost			
Total cost	74,680 €	25,238 €	42,481 €
Amortized cost	19,969 €	11,015 €	5697 €
Amortized cost per cow^a^	27.58 €	44.59 €	72.11 €
Biosecurity section (with amortization)			
Animal introduction	2880 €	4524 €	−€
Farm access by visitors and vehicles	3128 €	461 €	1583 €
Feed storage and water treatment	5493 €	3706 €	1711 €
Pest control	8467 €	2325 €	2403 €

*Note:* The current biosecurity cost is presented both in aggregate form and by section.

^a^Both lactating and dry cows were considered.

### 3.2. Risk of BVD Introduction and Associated Economic Impact

Under the existing biosecurity conditions, the estimated probability of BVD introduction was 2.66% (95% CI: 0.99–5.67), 15.70% (95% CI: 10.1–21.5), and 0.36% (95% CI: 0.15–0.73) for farms A, B, and C, respectively. Across the three farms, pathways associated with visitors and vehicles contributed the greatest risk of pathogen introduction. Consequently, most of the biosecurity measures recommended by the model targeted these pathways.

The economic impact of a potential BVD outbreak was estimated using the average results from the first 5 years of simulation. Compared with the BVD‐free scenario, the outbreak scenario reduced the average GM by −6.0%, −5.7%, and −5.9% on farms A, B, and C, respectively. Additional details regarding the SimHerd outputs are available in Supporting Information Annex 2 (Tables 1.S2–4.S2). The estimated probabilities of BVD introduction and the corresponding outbreak costs are summarized in Table 5.

**Table 5:** Risk of BVD introduction under different biosecurity scenarios and farm‐level costs of a potential BVD outbreak.

Farm	Scenarios	Probability of introduction of BVD (%)^a^	BVD outbreak cost at farm level and relative reduction in the gross margin
Farm A	Current level	2.66 (0.99–5.67)	−153,871 € (−6.0%)
	Provide boots to all drivers	1.55 (0.58–3.31)	
	Provide clothing to visitors	2.54 (0.94–5.41)	
	Provide boots to all visitors	2.55 (0.95–5.43)	

Farm B	Current level	15.7 (10.1–21.5)	−46,386 € (−5.7%)
	No shared transport	3.67 (2.36–5.03)	
	Provide boots to all drivers	14.4 (9.27–19.73)	
	Provide clothing to visitors	15.57 (10.02–21.32)	

Farm C	Current level	0.36 (0.15–0.73)	−8977 € (−5.9%)
	Provide boots to all drivers	0.22 (0.09–0.45)	
	Provide boots to all visitors	0.33 (0.14–0.67)	
	Provide clothing to visitors	0.33 (0.14–0.67)	

^a^Monte Carlo simulations are provided as median (50th percentile) with 95% confidence interval.

### 3.3. Decision Analysis on Biosecurity Improvements

Decision trees were developed for each farm to identify the most cost‐effective biosecurity improvement. The analysis compared the EMVs associated with the current biosecurity situation and alternative scenarios incorporating additional measures recommended by the risk assessment model. A summary of the EMV differences for all scenarios evaluated across the three farms is presented in Table 6.

**Table 6:** Summary of the pay‐off tables resulting from the decision analysis for the three farms included in the study.

Farm	Current risk level (%)	Scenario	Risk reduction (%)	Differences in EMV relative to the current biosecurity level^a^	
				Absolute change in EMV (€)	Relative change in EMV (%)
Farm A	2.7	Provide boots to all drivers^b^	41.6	1680 (608; 3607)	+7.0
		Provide clothing to visitors	4.6	162 (44; 375)	+0.7
		Provide boots to all visitors	4.3	152 (41; 351)	+0.6

Farm B	15.7	No shared transport	76.6	381 (−1609; 2442)	+2.1
		Provide boots to all drivers^b^	8.3	576 (362; 798)	+3.1
		Provide clothing to visitors	0.8	33 (12; 55)	+0.2

Farm C	0.4	Provide boots to all drivers	38.2	0 (−7; 13)	0
		Provide boots to all visitors	8.9	−22 (−23; −19)	−0.4
		Provide clothing to visitors	8.6	−24 (−25; −21)	−0.4

^a^The values presented correspond to the difference in EMV compared to the current biosecurity level, considering the average, minimum, and maximum risk of BVD introduction on the farm calculated by the risk analysis model. EMV (expected monetary value) is expressed as an expected economic outcome (negative values indicate costs); therefore, higher EMV values represent lower expected economic losses.

^b^Most cost‐effective biosecurity measure identified for the farm (i.e., scenario with the highest EMV).

For Farm A, the recommended interventions were the provision of boots for drivers entering animal areas, farm‐specific clothing for visitors in contact with animals, and dedicated boots for those visitors. Among these alternatives, providing boots to drivers resulted in the highest EMV and therefore represented the most cost‐effective option, with an improvement of €1680 compared with the current situation.

The same procedure was applied to Farms B and C. For Farm B, providing boots to drivers was again identified as the most favorable strategy, generating an EMV increase of €576. For Farm C, however, none of the evaluated measures improved the economic outcome relative to the biosecurity practices already implemented. Detailed payoff tables for all farms are provided in Supporting Information Annex 3 (Tables 1.S3–3.S3).

### 3.4. Sensitivity Analysis

The results of the decision tree model were robust across the range of values evaluated. Increasing the three key input parameters (probability of BVD introduction, outbreak cost, and biosecurity implementation cost) did not alter the preferred decision, defined as the scenario with the highest EMV. Across all sensitivity analyses, providing boots to drivers remained the strategy associated with the highest EMV.

When the probability of BVD introduction was increased to 50%, the EMV of the “Provide boots to all drivers” scenario was 33% higher than that of the current situation (EMV: −64,906.56 € vs. −96,904.09 €). The second most favorable option, providing protective clothing to visitors, improved the EMV by 3.6% relative to the current scenario (EMV: −93,387.78 € vs. −96,904.09 €), suggesting that both measures would be economically advantageous under higher introduction risk.

When the cost of a BVD outbreak was doubled, the “Provide boots to all drivers” strategy produced an EMV that was 12% higher than that of the current situation (EMV: −24,774.99 € vs. −28,159.64 €). This corresponds to an increase of 5% points compared with the original difference observed under baseline outbreak costs (7%). Likewise, doubling the cost of biosecurity implementation did not change the ranking of strategies. Under this scenario, the EMV of the “Provide boots to all drivers” option remained 3.8% higher than that of the current situation (EMV: −42,377.34 € vs. −44,032.70 €), although this represented a reduction of 3.2 percentage points from the initial 7% advantage.

Overall, the sensitivity analysis indicated that providing boots to drivers remained the most cost‐effective strategy under all conditions evaluated. Detailed sensitivity analysis results are available in Supporting Information Annex 3 (Tables 4.S3–6.S3).

## 4. Discussion

The findings of this study illustrate the potential economic value of external biosecurity measures, particularly those aimed at reducing the likelihood of BVD introduction. BVD was used as the reference disease because extensive quantitative evidence is available regarding its effects on production performance in dairy cattle, making it a suitable model for estimating outbreak‐related losses [18, 25, 27, 28, 32].

Previous studies have highlighted the importance of demonstrating the benefits of biosecurity to encourage adoption at the farm level [8, 33]. Farmers often perceive biosecurity investments as a cost without clearly recognizing their potential economic return. In this context, providing objective information on the expected benefits of preventive measures may facilitate informed decision‐making. Such information may also support veterinarians in their advisory role as they are commonly considered among the most trusted and influential sources of guidance for farmers [34, 35].

Most studies addressing the economics of biosecurity have relied either on case‐specific evaluations or on tools focusing exclusively on economic or epidemiological aspects. In contrast, the framework presented here combines farm production data, economic performance, pathogen introduction risk, outbreak consequences, and implementation costs within a single decision‐analysis approach. Although the framework relies on simulation models and therefore incorporates uncertainty and simplifications that may not fully capture real farm conditions [36–38], it provides a useful basis for illustrating the potential benefits of biosecurity measures at the farm level and may serve as a practical decision‐support and educational tool.

The parameters used within the framework were tailored to the characteristics and context of each farm. As a result, the approach can be adapted to different production systems and geographical settings through the use of context‐specific inputs. In addition, the modular design of the framework allows individual components to be applied independently, providing flexibility according to the objectives of the user. Decision analysis also proved to be a practical and straightforward approach for identifying cost‐effective biosecurity strategies. Similar approaches have previously been applied successfully in studies addressing animal health and farm‐level decision‐making [39, 40].

The results of the decision tree analysis suggest that relatively inexpensive biosecurity measures, such as the provision of farm‐specific boots or clothing, can offer the most favorable cost‐effectiveness under certain farm conditions. For example, on Farm B, the use of dedicated transport was identified as the measure with the greatest impact on risk reduction, decreasing the probability of BVD introduction by an estimated 77%. However, the higher implementation cost associated with this measure reduced its economic attractiveness. Consequently, providing boots to drivers resulted in a higher EMV, indicating lower expected economic losses once both the probability of disease introduction and the associated costs were considered.

It should be acknowledged that the estimates of both biosecurity costs and risk reduction assumed the correct implementation of the proposed measures. However, previous studies have shown that successful adoption of biosecurity practices depends not only on technical recommendations but also on farmers’ attitudes and behaviors [8]. Factors such as limited confidence in the measures, inconvenience, or difficulties associated with their practical implementation may reduce compliance and effectiveness [8, 41–43].

In addition, the estimated reduction in the probability of BVD introduction for each measure was obtained from a quantitative risk assessment model. Therefore, the effectiveness and economic performance of individual biosecurity measures should be interpreted within the specific context in which they are applied, as both may vary according to existing biosecurity levels and other farm‐specific factors.

Furthermore, demonstrating the potential economic benefits of biosecurity measures is only one of the factors influencing their adoption. Previous studies have identified additional barriers that may limit implementation, including time constraints, perceived practicality, and farmers’ personal beliefs [8, 9, 11]. Although economic incentives may encourage the uptake of biosecurity measures in some situations, other behavioral and contextual factors may also play an important role. Consequently, the decision‐making process surrounding biosecurity is more complex than the economic considerations evaluated in the present study, which should be regarded as a limitation.

The estimated biosecurity cost varied substantially among the farms included in the study, ranging from €27.6 to €72.1 per cow on an annualized basis. The highest cost per cow was observed in the smallest farm, suggesting that achieving a comparable level of biosecurity may require a proportionally greater economic investment in smaller operations. From a policy perspective, these results indicate that farms with more limited financial resources may encounter additional barriers when attempting to adopt new biosecurity measures. In such cases, support mechanisms such as targeted subsidies or incentive programmes aimed at improving biosecurity standards could facilitate their implementation [15, 44]. Comparisons of biosecurity costs between studies should nevertheless be interpreted with caution, as published estimates are often based on different production systems, geographic regions, and sets of biosecurity measures [17, 45].

In the present study, the analysis was restricted to the probability of BVD introduction and the economic consequences of a BVD outbreak. The estimated outbreak cost ranged from €114 to €213 per cow and accounted for the effects of increased mastitis risk, lower conception rates, and reduced milk production. These estimates are consistent with values previously reported in a review of the economic impact of BVD [46].

Nevertheless, the results should be interpreted in light of the study design. The framework was evaluated using data from only three dairy farms and was primarily intended as a proof‐of‐concept application. Farm‐specific characteristics, including herd size, production performance, and cost structure, may influence both the estimated outbreak costs and the relative cost‐effectiveness of biosecurity measures, thereby limiting the direct extrapolation of the results to other production systems.

Biosecurity measures can also contribute to preventing the introduction and spread of many other endemic and exotic diseases [47, 48]. Therefore, the overall value of the proposed framework could be greater if additional pathogens were incorporated into the analysis, particularly diseases with a low probability of occurrence but potentially severe economic consequences, such as transboundary animal diseases. Under such circumstances, the general analytical framework could be maintained while adapting the epidemiological and economic parameters used to estimate the introduction risk and outbreak costs.

In some farms, the measures recommended by the model did not outperform the biosecurity practices already in place, which may reflect the specific production characteristics and disease risk profile of those farms. However, the inclusion of additional diseases would likely increase the differences in EMVs among alternative strategies. Finally, the present analysis focused on external biosecurity measures and did not explicitly incorporate other BVD prevention and control tools, such as surveillance programmes or vaccination. Including these interventions could modify both the probability of disease introduction and the resulting economic outcomes.

The biosecurity cost calculator developed for this study does not currently account for certain structural or management‐related biosecurity measures, mainly because of the challenges associated with their quantification. In addition, some of the items included in the calculator may provide benefits beyond biosecurity, such as improvements related to feed quality or water hygiene (e.g., silage storage and water sanitization) [49]. The measures currently incorporated into the tool are predominantly related to external biosecurity. Future developments should therefore consider the inclusion of internal biosecurity measures and their potential effect on pathogen transmission within the farm. Furthermore, the calculator requires periodic updates if it is to be applied over time or in different geographical contexts, as the current version only includes prices from suppliers operating in Spain. Nevertheless, the structure of the tool allows prices and materials to be modified when necessary, and future versions could incorporate automated price updates.

Future studies should continue improving the estimation of the economic benefits associated with biosecurity investments at the farm level. In addition, further research is needed to evaluate how communication of the economic value and effectiveness of biosecurity measures influences farmers’ decision‐making and the adoption of these practices.

## 5. Conclusions

The framework presented in this study enabled the ranking of biosecurity measures according to their cost‐effectiveness, supporting farm‐level decision‐making within the specific risk context of each farm. By integrating biosecurity costs, disease‐introduction risk, and outbreak‐related economic losses, the approach provides a practical basis for comparing alternative biosecurity strategies. The biosecurity cost calculator and the associated analytical framework may assist farmers and advisors in identifying measures that achieve an appropriate balance between implementation costs and risk reduction.

As the framework was evaluated as a proof of concept using three dairy farms and a single disease, additional studies involving different pathogens, production systems, and geographical settings are needed to further assess its applicability and robustness.

## Author Contributions

Conceptualization, funding acquisition: Alberto Allepuz, Jordi Casal, and Giovanna Ciaravino. Methodology: Fernando Duarte, Alberto Allepuz, Jordi Casal, and Giovanna Ciaravino. Validation, writing – original draft preparation: Fernando Duarte, Alberto Allepuz, Arnau Àlvarez, Jordi Casal, Natalia Ciria, Bodil Højlund, Jehan Ettema, and Giovanna Ciaravino. Formal analysis, investigation, writing – review and editing, data curation, visualization: Fernando Duarte, Alberto Allepuz, and Giovanna Ciaravino. Supervision: Alberto Allepuz and Giovanna Ciaravino.

## Funding

This research project was funded by BioRisk (MCIN/AEI, Grant PID2020‐118302RB‐I00). This work was funded by the European Union under the Horizon Europe Grant 101083923 (BIOSECURE). Views and opinions expressed are, however, those of the author(s) only and do not necessarily reflect those of the European Union or the European Research Executive Agency (REA). Neither the European Union nor the granting authority can be held responsible for them. The first author is financed by the Chilean National Agency for Research and Development (ANID)/Scholarship Program/DOCTORADO BECAS CHILE/(Grant 2020–72210236).

## Disclosure

All authors have read and agreed to the published version of the manuscript.

## Ethics Statement

This work has been approved by the Ethics Committee of the Autonomous University of Barcelona—Approval Number CEEAH 6188.

## Conflicts of Interest

The authors declare no conflicts of interest.

## Supporting Information

Additional supporting information can be found online in the Supporting Information section.

## Supporting information


**Supporting Information** Annex 1: Cost estimation of biosecurity measures, Figure 1.S1, Table 1.S1, and Table 2.S1; Annex 2: Outputs obtained from the SimHerd simulation, Table 1.S2, Table 2.S2, Table 3.S2, and Table 4.S2; and Annex 3: Decision analysis on the most cost‐effective biosecurity improvements, Table 1.S3, Table 2.S3, Table 3.S3, Table 4.S3, Table 5.S3, and Table 6.S3.

## Data Availability

All other data will be provided upon request.
