# Supplementary material for: Tailoring Biosecurity Advice in Dairy Cattle Farms: Decision Analysis to Estimate the Most Cost‐Effective Option
Source: Transbound Emerg Dis. 2026 Aug 31;2026:1472113. doi: 10.1155/tbed/1472113 (PMC13528622; doi:10.1155/tbed/1472113)
Supplement: Supplementary file 1 [file TBED-2026-1472113-s001.docx]

**Supporting Information**

**Annex 1 - Cost estimation of biosecurity measures.**

**Figure 1.S1. Survey used for data collection.**

**Table 1.S1.** **List of items used to estimate the cost of biosecurity measures considered in the cost estimation.**

| **Data price** | **Cost type** | **Useful life** | **Units** | **Annual frequency** | **Quantity** | **Price 1** | **Price 2** | **Price 3** | **Mean/**  **Total** | **Source**  **1** | **Source**  **2** | **Source**  **3** |
| --- | --- | --- | --- | --- | --- | --- | --- | --- | --- | --- | --- | --- |
| **Perimeter fencing** |  |  |  |  |  |  |  |  |  |  |  |  |
| Concrete block wall 2.5m materials | Structural | 40 | Meter |  |  |  |  |  | 69.54 € |  |  |  |
| Materials | Variable |  | Meter |  |  | 23.27 € |  |  | 23.27 € |  |  |  |
| Labour + tools | Variable |  | Hour/meter |  |  | 46.27 € |  |  | 46.27 € | [Source1](http://www.generadordeprecios.info/obra_nueva/calculaprecio.asp?Valor=4\|0_0_0_0_0\|1\|UVM010\|uvm_010:c4_0_200c16_0_1c5_0_1_5_5_1#gsc.tab=0) |  |  |
| Brick wall 2.5m materials | Structural | 40 | Meter |  |  |  |  |  | 131.90 € |  |  |  |
| Materials | Variable |  | Meter |  |  | 48.45 € |  |  | 48.45 € |  |  |  |
| Labour + tools | Variable |  | Hour/meter |  |  | 83.45 € |  |  | 83.45 € | [Source1](http://www.generadordeprecios.info/obra_nueva/calculaprecio.asp?Valor=2.5\|0_0_0_0\|1\|UVM010\|uvm_010:c4_0_50c16_0_1c5_0_1_5_5_1#gsc.tab=0) |  |  |
| Concrete wall 2.5m materials | Structural | 40 | Meter |  |  |  |  |  | 167.84 € |  |  |  |
| Materials | Variable |  | Meter |  |  | 150.69 € |  |  | 150.69 € |  |  |  |
| Labour + tools | Variable |  | Hour/meter |  |  | 17.15 € |  |  | 17.15 € | [Source1](http://www.generadordeprecios.info/obra_nueva/calculaprecio.asp?Valor=2.5%20\|0_0\|1\|UVM020\|uvm_020:_0_0_50_0_500c4_0_1c3_0_1_0_0_2_4_3_3_0_1c3_0_5_0_2_0#gsc.tab=0) |  |  |
| Twisted wire mesh 2m (3cm mesh spacing) | Structural | 20 |  |  |  |  |  |  | 31.24 € |  |  |  |
| Materials | Variable |  | Meter |  |  | 25.40 € |  |  | 25.40 € | [Source1](http://www.generadordeprecios.info/obra_nueva/calculaprecio.asp?Valor=7\|0_0\|0\|UVT010\|uvt_010:_0_0_5_0_0_0_2_0#gsc.tab=0) |  |  |
| Labour + tools | Variable |  | Hour/meter |  |  | 5.84 € |  |  | 5.84 € |  |  |  |
| Double fencing (multiplier by quantity) | Multiplier |  |  |  | 2 |  |  |  |  |  |  |  |
| Fence maintenance/  inspection |  |  | % initial investment over useful life | 1 | 0.1 |  |  |  |  |  |  |  |
| **Vehicle entry** |  |  |  |  |  |  |  |  |  |  |  |  |
| Disinfection arch | Fixed | 10 | Un |  | 1 | 984.54 € | 4,407.48 € |  | 2,696.01 € | [Source1](https://ventas.suministrosganaderos.com/index.php?route=product/category&path=491) | [Source2](https://www.schippersweb.com/ms-arco-de-desinfeccion-2-0-kit-completo-2509441.html) |  |
| Disinfectant solution for footbath | Variable | 1 | Lt |  | 17.94 | 145.56 € | 498.33 € |  | 321.95 € | [Source1](https://www.schippersweb.com/ms-t-t-cleaner-2-0-M2509915.html) | [Source2](https://www.schippersweb.com/ms-megades-para-M2505200.html?gad_source=1&gclid=CjwKCAiA0bWvBhBjEiwAtEsoW8Z_QbHvv1FcSkQCOYYpH3FQs-LSXXxtKMniilob06qvuSyuqsj44xoCr6sQAvD_BwE) |  |
| **Visitors change area** |  |  |  |  |  |  |  |  |  |  |  |  |
| Latex gloves | Variable |  | Un | 260 | 100 | 6.34 € | 8.47 € | 9.14 € | 7.98 € | [Source1](https://www.schippersweb.com/ms-guantes-latex-empolvado-p-100-M5609597.html) | [Source2](https://www.333shop.com/es/shop/product/guantes-de-latex-100-uds-sin-polvo-6378/10980#/500-talla_manos-l) | [Source3](https://www.reysan.com/guante-surey-nitrilo-premium.aspx) |
| Disposable polypropylene overall | Variable |  | Un | 260 | 1 | 3.36 € | 1.54 € | 2.36 € | 2.42 € | [Source1](https://www.schippersweb.com/ms-mono-desechable-azul-M5603841.html) | [Source2](https://acortar.link/H6Ox8R) | [Source3](https://www.reysan.com/mono-propileno-plus.aspx) |
| Disposable plastic boot covers | Variable |  | Un | 260 | 50 | 10.05 € | 13.35 € | 13.31 € | 12.24 € | [Source1](https://www.schippersweb.com/bota-plastico-desechable-0-07-mm-p-50-5503305.html) | [Source2](https://www.333shop.com/es/shop/product/369-bota-de-plastico-desechable-con-goma-007-mm-50-uds-5356) | [Source3](https://www.prolaboral.com/es/2201-cubrebotas-desechable-polietileno.html) |
| Time in change area per day per worker (hours) | Variable |  |  |  |  |  |  |  |  |  |  |  |
| Plastic tub | Fixed | 2 | Un |  | 1 | 9.55 € | 22.91 € |  | 16.23 € | [Source1](https://www.schippersweb.com/ms-pediluvio-pequeno-12-l-6403089.html) | [Source2](https://www.333shop.com/es/shop/product/3717-pediluvio-60-x-40-x-9-cm-7494?gclid=CjwKCAiA3pugBhAwEiwAWFzwdTxNRmDBF0npE6HlLYcP7nKApyKgby1ygbjd-NnpamTLKeb1Z_DU4BoC3HQQAvD_BwE) |  |
| Disinfection mat | Fixed | 2 | Un |  | 1 | 57.91 € | 47.01 € | 51.97 € | 52.30 € | [Source1](https://www.schippersweb.com/pediluvio-azul-M2509755.html) | [Source2](https://www.333shop.com/es/shop/product/206-pediluvio-alfombra-desinfectante-333-90x60x4-cm-5264) | [Source3](https://www.reysan.com/alfombrilla-desinfectante-calzado-antideslizante-uso-profesional.aspx) |
| Disinfectant solution | Variable |  | Lt |  | 17.94 | 145.56 € |  |  | 145.56 € | [Source1](https://www.schippersweb.com/ms-megades-oxy-M2509801.html) |  |  |
| Concentrated disinfectant for footbath | Variable |  | Lt |  | 20 | 99.00 € |  |  | 99.00 € | [Source1](https://dimercos.com/desinfectantes-para-granjas/24-101-desinfectante-g-10.html#/59-formatos-20l) |  |  |
| Automatic boot cleaner | Fixed | 5 | Un |  | 1 | 120.00 € | 148.00 € | 293.03 € | 187.01 € | [Source1](https://www.ganaderiaymascotas.com/comprar/accesorios-para-granja/limpiabotas-inox) | [Source2](https://www.schippersweb.com/limpiador-de-botas-acero-inoxidable-2505850.html) | [Source3](https://tiendaganadera.com/cepillo-limpiabotas/) |
| Farm-specific boots | Variable | 2 | Pair |  | 1 | 38.48 € | 22.54 € | 12.90 € | 24.64 € | [Source1](https://www.333shop.com/es/shop/product/botas-de-seguridad-de-pvc-delta-plus-iron-s5-9066/13936#/225-talla_pie-39) | [Source2](https://acortar.link/7f8bqN) | [Source3](https://acortar.link/OvgUP7) |
| Time in change area per day (per worker) in hours | Variable | 1 | Hour | 260 | 0.17 |  |  |  |  |  |  |  |
| **Signage** |  |  |  |  |  |  |  |  |  |  |  |  |
| No entry sign | Fixed | 3 | Un |  | 1 | 14.75 € |  |  | 14.75 € | [Source1](https://www.schippersweb.com/senal-de-advertencia-prohibido-el-paso-4100130.html) |  |  |
| **Access (gates)** |  |  |  |  |  |  |  |  |  |  |  |  |
| Lifting barrier | Fixed | 10 | Un |  | 1 | 1,014.89 € |  |  | 1,014.89 € | [Source1](https://generadordeprecios.info/obra_nueva/Urbanizacion_interior_de_la_parcela/Mobiliario_urbano/Protecciones_peatonales/Barrera_levadiza_peatonal.html) |  |  |
| Manual swing gate | Fixed | 20 | Un |  | 1 | 2,495.41 € |  |  | 2,495.41 € | [Source1](https://generadordeprecios.info/remote.asp?Command=0,browse,idioma:0%5bselector:Valor=0\|0\|0\|UVP010\|uvp_010:_1_0_0_0_0_0_0_80_0%5bn:164489%5b) |  |  |
| Automatic swing gate | Fixed | 20 | Un |  | 1 | 3,667.06 € |  |  | 3,667.06 € | [Source1](https://generadordeprecios.info/remote.asp?Command=0,browse,idioma:0%5bselector:Valor=1\|0_0\|1\|UVP010\|uvp_010:_0_0_0_0_0_0_20_80_0%5bn:179727%5b) |  |  |
| Manual sliding gate | Fixed | 20 | Un |  | 1 | 2,962.39 € |  |  | 2,962.39 € | [Source1](https://generadordeprecios.info/remote.asp?Command=0,browse,idioma:0%5bselector:Valor=1_0_1\|0\|1\|UVP010\|uvp_010:_0_1_0_0_0_0_40_80_0_0%5bn:183616%5b) |  |  |
| Automatic sliding gate | Fixed | 20 | Un |  | 1 | 3,949.02 € |  |  | 3,949.02 € | [Source1](https://generadordeprecios.info/remote.asp?Command=0,browse,idioma:0%5bselector:Valor=1\|0_0\|1\|UVP010\|uvp_010:_0_1_0_0_0_0_40_80_0_0%5bn:183770%5b) |  |  |
| **Mortality disposal** |  |  |  |  |  |  |  |  |  |  |  |  |
| Concrete surface 9m² | Structural | 20 | Un |  | 1 | 217.05 € |  |  | 217.05 € | [Source1](https://generadordeprecios.info/remote.asp?Command=0,config,idioma:0%5bn:245801%5b) |  |  |
| Mortality fencing (9m² = 14.4 linear metres) | Structural | 20 | Un |  | 1 | 449.86 € |  |  | 449.86 € |  |  |  |
| Mortality handling per cow/year (collection company) | Variable |  | Cow/year |  |  | 6.70 € |  |  | 6.70 € | Source1 |  |  |
| **Feed storage** |  |  |  |  |  |  |  |  |  |  |  |  |
| Silo – 7,000 kg | Fixed | 10 | Un |  | 1 | 1,531.09 € | 2,317.15 € |  | 1,924.12 € | [Source1](https://ventas.suministrosganaderos.com/index.php?route=product/product&path=1900_1901&product_id=1903003) | [Source2](https://tiendaganadera.com/silos-para-pienso/) |  |
| Silo – 10,000 kg | Fixed | 10 | Un |  | 1 | 1,780.80 € | 1,914.22 € |  | 1,847.51 € | [Source1](https://ventas.suministrosganaderos.com/index.php?route=product/product&path=1900_1901&product_id=1901005&sort=p.price&order=ASC&limit=100) | [Source2](https://tiendaganadera.com/SILO-METALICO-CHAPA-ONDULADA-10.120-Kg/) |  |
| Silo – 17,000 kg | Fixed | 10 | Un |  | 1 | 2,227.20 € | 2,524.06 € |  | 2,375.63 € | [Source1](https://ventas.suministrosganaderos.com/index.php?route=product/product&path=1900_1901&product_id=1903008&sort=p.price&order=ASC&limit=100) | [Source2](https://tiendaganadera.com/SILO-METALICO-CHAPA-ONDULADA-17.160-Kg/) |  |
| Feed bin with lid (500/600L) | Fixed | 10 | Un |  | 1 | 390.00 € | 784.96 € |  | 587.48 € | [Source1](https://hipisur.com/verDetalle.php?utm_src=feed&pid=8458&gclid=Cj0KCQjwxMmhBhDJARIsANFGOStdu8DlW1Odm7FJ5_H8lWFC_4WNfy8tm6f1RH7ruPZBB2VnxTNgDGQaAt51EALw_wcB) | [Source2](https://grupacer.com/tienda/contenedores-para-pienso/5246-contenedor-para-grano-600-l.html) |  |
| Pallets (up to 750 kg) | Fixed | 3 | Un |  | 1 | 16.32 € | 14.51 € |  | 15.42 € | [Source1](https://www.rotomshop.es/palet-madera-1200x1000x146mm-usado.html?source=googlebase&gclid=CjwKCAiAu5agBhBzEiwAdiR5tPkpFzh886pRtthPV194JqhxtQZA-R1AtkleTGzjBs_Ny1N3pAToQxoCDLgQAvD_BwE) | [Source2](https://www.rotomshop.es/palet-intermedio-1200x800x126mm-usado.html?source=googlebase&gclid=CjwKCAiA3pugBhAwEiwAWFzwdaFa9wuu1aDmnXG8l2hF8vCdm_wdhGWdF1nQnfwelyZZfKw5tVs4bhoCazEQAvD_BwE) |  |
| Water Treatment |  |  |  |  |  |  |  |  |  |  |  |  |
| Hydrogen peroxide 50% | Variable |  | Lt |  | 1195 | 2,977.81 € |  |  | 2,977.81 € | [Source1](https://dimercos.com/desinfectantes/148-516-peroxido-de-hidrogeno-50.html#/190-formatos-1195kg) |  |  |
| 220V dosing pump | Fixed | 5 | Un |  | 1 | 325.00 € |  |  | 325.00 € | [Source1](https://www.ganaderiaymascotas.com/comprar/dosificacion-de-medicamentos-peroxidos-cloro/dosificador-peroxido-proporcional-220-v-) |  |  |
| Hydrogen peroxide test strips | Variable |  | Un |  | 100 | 64.98 € | 51.02 € |  | 58.00 € | [Source1](https://www.333shop.com/es/shop/product/117-quantofix-peroxido-100-5208) | [Source2](https://www.manomano.es/p/aquachek-peroxide-tester-3-en-1-38890878) |  |
| Sodium hypochlorite | Variable |  | Lt |  | 1036 | 963.48 € |  |  | 963.48 € | [Source1](https://dimercos.com/desinfectantes/22-42-cuhigen-cloro-hipoclorito-sodico.html#/28-formatos-1036_kg) |  |  |
| Chlorine test strips | Variable |  | Un |  | 50 | 23.00 € |  |  | 23.00 € | [Source1](https://dimercos.com/medidores/122-tiras-reactivas-de-cloro.html) |  |  |
| **Pest Control – Physical Measures** |  |  |  |  |  |  |  |  |  |  |  |  |
| Mouse trap clamp | Fixed | 1 | Un | 1 | 1 | 2.48 € | 1.52 € | 2.99 € | 2.33 € | [Source1](https://www.333shop.com/es/shop/product/2671-ratonera-metalica-grande-6490) | [Source2](https://tiendaganadera.com/cepo-ratones/) |  |
| Sticky trap for rats and mice (33 × 21 cm / 135 g glue) | Fixed | 1 | Un | 1 | 1 | 2.91 € | 3.91 € |  | 3.41 € | [Source1](https://www.schippersweb.com/placa-adhesiva-ratas-y-ratones-2408083.html) | [Source2](https://www.333shop.com/es/shop/product/4633-rodi-clac-pegamento-tubo-de-135-g-8385) |  |
| Adhesive fly and mosquito tape (10 m × 25 cm / 30 sticky sheets) | Fixed | 1 | Un | 12 | 1 | 14.06 € | 28.36 € |  | 21.21 € | Source1 | [Source2](https://www.333shop.com/es/shop/product/2612-placas-adhesivas-professional-30-6432) |  |
| Cockroach glue trap | Variable |  | Un | 12 | 5 | 6.53 € | 8.32 € |  | 7.43 € | Source1 | [Source2](https://www.333shop.com/es/shop/product/4639-blatclac-trampas-engomadas-con-cebo-para-cucarachas-8391) |  |
| Fly and wasp trap | Fixed | 1 | Un | 12 | 1 | 14.84 € | 14.21 € |  | 14.53 € | [Source1](https://www.schippersweb.com/atrapa-mosca-y-avispa-2309852.html) | [Source2](https://tiendaganadera.com/trampa-moscas/) |  |
| Sealant paste for openings | Variable |  | mL | 1 | 300 | 13.07 € | 12.33 € |  | 12.70 € | Source1 | [Source2](https://www.333shop.com/es/shop/product/3976-masilla-para-roedores-mousestop-300-ml-7742) |  |
| **Pest Control – Repellents** |  |  |  |  |  |  |  |  |  |  |  |  |
| Animal and rodent repellent (500 ml, with 2 dispensers) / ultrasonic coverage up to 500 m² | Variable |  | Un |  | 1 | 88.95 € | 94.16 € |  | 91.56 € | [Source1](https://www.schippersweb.com/ms-tupoleum-kit-de-inicio-mini-500-ml-2400064.html) | [Source2](https://tiendaganadera.com/AHUYENTADOR-DE-RATONES-CUCARACHAS-Y-MURCIELAGOS-POR-ULTRASONIDO-500-m2/) |  |
| Ultrasonic bird and pigeon repellent (500 m² coverage) | Variable |  | Un |  | 1 | 118.10 € |  |  | 118.10 € | [Source1](https://tiendaganadera.com/AHUYENTADOR-DE-PALOMAS-Y-OTRAS-AVES-POR-DISPAROS-9-m./) |  |  |
| Wild boar repellent | Variable |  | Un |  | 40 | 29.61 € |  |  | 29.61 € | [Source1](https://www.333shop.com/es/shop/product/3241-jabalex-repelente-para-jabalies-2-kg-7035) |  |  |
| **Pest Control – Chemical Rodent Control** |  |  |  |  |  |  |  |  |  |  |  |  |
| Rodenticide bait | Variable |  | Un |  | 6 | 17.07 € |  |  | 17.07 € | Source1 |  |  |
| Rodent bait station (for rats and mice) | Variable |  | Un | 2 | 1 | 4.55 € | 6.86 € |  | 5.71 € | [Source1](https://www.333shop.com/es/shop/product/638-portacebos-de-plastico-para-ratas-y-ratones-5554) | [Source2](https://tiendaganadera.com/portacebos-raticida/) |  |
| 5L sprayer for phytosanitary products | Variable |  | Un |  | 1 | 49.00 € |  |  | 49.00 € | [Source1](https://www.schippersweb.com/gloria-pulverizador-por-presion-prima-5-0809662.html) |  |  |
| Time spent checking traps/bait (hours) | Variable |  | Hour | 12 | 2 |  |  |  |  |  |  |  |
| **Perimeter and Wildlife Control** |  |  |  |  |  |  |  |  |  |  |  |  |
| Electric fence energiser | Fixed | 5 | Un |  | 1 | 263.57 € | 277.09 € |  | 270.33 € | [Source1](https://ventas.suministrosganaderos.com/index.php?route=product/product&path=7300_7301&product_id=7301040) | [Source2](https://tiendaganadera.com/PASTOR-ELECTRICO-ZAKO-PILA-RED-CON-PILA-DE-55-A-h./) |  |
| Conductive tape | Variable |  | Mt |  | 200 | 35.00 € | 31.00 € | 25.95 € | 30.65 € | [Source1](https://www.pastorelectrico.com/cables-cintas-conductoras/cintas-conductoras/cinta-conductora-veloz-40-mm-200-metros) | [Source2](https://www.pastorelectrico.com/cables-cintas-conductoras/cintas-conductoras/cinta-conductora-superveloz-20-mm-200-metros) | [Source3](https://www.pastorelectrico.com/cables-cintas-conductoras/cintas-conductoras/cinta-conductora-neon-gama-eco-40-mm-200-metros) |
| **Feed and Water Supply for Extensive Systems** |  |  |  |  |  |  |  |  |  |  |  |  |
| Elevated drinkers | Fixed | 10 | Un |  | 1 | 392.18 € | 174.02 € |  | 283.10 € | [Source1](https://acortar.link/H9nYOc) | [Source2](https://www.mallasgalbis.es/es/material-vacuno/291-684-bebedero-de-vacas-galvanizado-con-boya.html#/13-longitud-2_m) |  |
| Covered raised feeder with protective bars | Fixed | 10 | Un |  | 1 | 800.00 € | 1.386.72 € | 621.32 € | 936.01 € | [Source1](https://www.milanuncios.com/otras-maquinarias-agricolas/comedero-1-70-x-1-70-33973018.htm) | [Source2](https://www.mallasgalbis.es/es/material-vacuno/298-705-comedero-vacuno-galvanizado-en-caliente.html#/13-longitud-2_m) | [Source3](https://www.mallasgalbis.es/es/material-vacuno/303-714-comedero-de-vacuno-rectangular.html#/258-con_arquillos-si/267-con_techo-si) |
| Separate drinkers for wildlife | Fixed | 10 | Un |  | 1 | 74.05 € |  |  | 74.05 € | [Source1](https://www.electrochannel.com/prefabricados-de-hormigon-y-tapas-de-fundicion/3995-bebedero-para-caza?gclid=Cj0KCQjwlPWgBhDHARIsAH2xdNcK4nkZ7Iex9KUFOLriPwhma90OuxaUavDUBgEIz0Bic-cGASmuz90aAk6mEALw_wcB) |  |  |
| Concrete surface for drinkers (per m²) | Structural | 15 | m2 |  | 1 | 11.45 € |  |  | 11.45 € | [Source1](http://www.generadordeprecios.info/obra_nueva/Revestimientos/Suelos_y_pavimentos/RSB_Bases_de_pavimentacion_y_grand/Base_de_mortero_de_cemento.html?mradio_00000C40=on) |  |  |
| **Hand Washing Station** |  |  |  |  |  |  |  |  |  |  |  |  |
| Hands-free sink (knee- or foot-operated) | Fixed | 10 | Un |  | 1 | 464.80 € | 343.99 € |  | 404.40 € | [Source1](https://www.schippersweb.com/fregadero-automatico-operado-por-la-rodilla-3609990.html) |  |  |
| Soap dispenser  (1.8 L) | Variable |  | Un |  | 1 | 25.44 € | 17.85 € |  | 21.65 € | [Source1](https://www.schippersweb.com/dispensador-de-jabon-ms-1-8-l-3609991.html) | [Source2](https://www.quirumed.com/es/lavamanos-de-pie-integral-de-dimensiones-reducidas-con-pulsador.html?gclid=CjwKCAiA3pugBhAwEiwAWFzwdUTrNRUFcjKqGsib6CifsTnzkgNNglXzWnwqMFwTierb7jzreb8w4RoCpkwQAvD_BwE) |  |
| Hand sanitiser | Variable |  | Lt |  | 1 | 8.96 € | 17.85 € |  | 13.41 € | [Source1](https://www.schippersweb.com/gel-de-manos-ms-skinclean-1-l-3609553.html) | [Source2](https://www.zambuonline.com/dosificador-industrial-jabon-gel-2-litros.html) |  |
| Pre-cut paper roll (180 m) | Variable |  | Un |  | 6 | 56.92 € |  |  | 56.92 € | [Source1](https://www.schippersweb.com/papel-2-capas-precortado-180m-x-22cm-p-6-1901513.html) | [Source2](https://acortar.link/IebUHj) |  |
| **Protective Clothing and Footwear** |  |  |  |  |  |  |  |  |  |  |  |  |
| Latex gloves | Variable |  | Un |  | 100 | 6.34 € | 8.47 € | 9.14 € | 7.98 € | [Source1](https://www.schippersweb.com/ms-guantes-latex-empolvado-p-100-M5609597.html) |  |  |
| Disposable polypropylene overall | Variable |  | Un |  | 1 | 3.36 € | 1.54 € | 2.36 € | 2.42 € | [Source1](https://www.schippersweb.com/ms-mono-desechable-azul-M5603841.html) | [Source2](https://www.333shop.com/es/shop/product/guantes-de-latex-100-uds-sin-polvo-6378/10980#/500-talla_manos-l) | [Source3](https://www.reysan.com/guante-surey-nitrilo-premium.aspx) |
| Disposable plastic boot covers | Variable |  | Un |  | 50 | 10.05 € | 13.35 € | 13.31 € | 12.24 € | [Source1](https://www.schippersweb.com/bota-plastico-desechable-0-07-mm-p-50-5503305.html) | [Source2](https://acortar.link/H6Ox8R) | [Source3](https://www.reysan.com/mono-propileno-plus.aspx) |
| Plastic tub | Fixed | 3 | Un |  | 1 | 9.55 € | 22.91 € |  | 16.23 € | [Source1](https://www.schippersweb.com/ms-pediluvio-pequeno-12-l-6403089.html) | [Source2](https://www.333shop.com/es/shop/product/369-bota-de-plastico-desechable-con-goma-007-mm-50-uds-5356) | [Source3](https://www.prolaboral.com/es/2201-cubrebotas-desechable-polietileno.html) |
| Disinfection mat | Fixed | 2 | Un |  | 1 | 57.91 € | 47.01 € | 51.97 € | 52.30 € | [Source1](https://www.schippersweb.com/pediluvio-azul-M2509755.html) | [Source2](https://www.333shop.com/es/shop/product/3717-pediluvio-60-x-40-x-9-cm-7494?gclid=CjwKCAiA3pugBhAwEiwAWFzwdTxNRmDBF0npE6HlLYcP7nKApyKgby1ygbjd-NnpamTLKeb1Z_DU4BoC3HQQAvD_BwE) |  |
| Automatic boot cleaner | Fixed | 5 | Un |  | 1 | 120.00 € | 148.00 € | 293.03 € | 187.01 € | [Source1](https://www.ganaderiaymascotas.com/comprar/accesorios-para-granja/limpiabotas-inox) | [Source2](https://www.333shop.com/es/shop/product/206-pediluvio-alfombra-desinfectante-333-90x60x4-cm-5264) | [Source3](https://www.reysan.com/alfombrilla-desinfectante-calzado-antideslizante-uso-profesional.aspx) |
| Disinfectant solution | Variable |  | Kg |  | 17.94 | 145.56 € |  |  | 145.56 € | [Source1](https://www.schippersweb.com/ms-megades-para-M2505200.html) | [Source2](https://www.schippersweb.com/limpiador-de-botas-acero-inoxidable-2505850.html) | [Source3](https://tiendaganadera.com/cepillo-limpiabotas/) |
| Reusable overall | Fixed | 2 | Un |  | 1 | 27.92 € | 24.75 € |  | 26.34 € | [Source1](https://www.schippersweb.com/ms-mono-cremallera-M5609595.html) | [Source2](https://www.masuniformes.com/buzo-trabajo-velilla-colores) |  |
| **Transport Disinfection** |  |  |  |  |  |  |  |  |  |  |  |  |
| Foaming cleaning product | Variable |  | Kg |  | 17.94 | 145.56 € |  |  | 145.56 € | [Source1](https://www.schippersweb.com/ms-megades-para-M2505200.html) |  |  |
| Foam application gun | Fixed | 3 | Un |  | 1 | 27.92 € | 24.75 € |  | 26.34 € | [Source1](https://www.schippersweb.com/ms-mono-cremallera-M5609595.html) | [Source2](https://www.masuniformes.com/buzo-trabajo-velilla-colores) |  |
| **Diagnostic Testing** |  |  |  |  |  |  |  |  |  |  |  |  |
| BVD (Bovine Viral Diarrhoea) | Variable |  | Un |  | 1 | 7.00 € | 9.50 € | 12.60 € | 9.70 € | [Source1](https://analiticaveterinaria.com/catalogo/catalogo-vacuno/) | [Source2](https://www.vithaslab.es/assets/doc/catlg-veterianaria-vithaslab-2020-21.pdf) | [Source3](https://allic.org/preus-sanitat-animal-2/) |
| IBR (Infectious Bovine Rhinotracheitis) | Variable |  | Un |  | 1 | 7.00 € | 9.50 € | 4.40 € | 6.97 € | [Source1](https://analiticaveterinaria.com/catalogo/catalogo-vacuno/) | [Source2](https://www.vithaslab.es/assets/doc/catlg-veterianaria-vithaslab-2020-21.pdf) | [Source3](https://allic.org/preus-sanitat-animal-2/) |
| Neospora | Variable |  | Un |  | 1 | 7.00 € | 11.00 € | 4.40 € | 7.47 € | [Source1](https://analiticaveterinaria.com/catalogo/catalogo-vacuno/) | [Source2](https://www.vithaslab.es/assets/doc/catlg-veterianaria-vithaslab-2020-21.pdf) | [Source3](https://allic.org/preus-sanitat-animal-2/) |
| Paratuberculosis | Variable |  | Un |  | 1 | 7.00 € | 8.00 € |  | 7.50 € | [Source1](https://analiticaveterinaria.com/catalogo/catalogo-vacuno/) | [Source2](https://www.vithaslab.es/assets/doc/catlg-veterianaria-vithaslab-2020-21.pdf) |  |
| **Sampling** |  |  |  |  |  |  |  |  |  |  |  |  |
| Time spent by vet and farmer on quarantine testing (hours) | Variable |  | Hour/  animal |  | 0.0167 |  |  |  |  |  |  |  |

**Table 2.S1.** **List of biosecurity measures included in the cost estimation.**

| Pathway of introduction | Biosecurity measure | Number of farm-level inputsᵃ |
| --- | --- | --- |
| Animal introduction | Quarantine | Number of animal entries (batches per year) |
|  |  | Number of animals entering per year (external source) |
|  |  | Does the farm have a quarantine facility? |
|  |  | Is the quarantine area within the farm perimeter? |
|  |  | Duration of quarantine (days) |
|  |  | Perimeter of quarantine area (m) |
|  |  | Frequency of access to quarantine area (daily) |
|  |  | Time spent daily in the quarantine pen (hours) |
|  |  | Number of workers entering the quarantine area |
|  |  | Type of fencing around quarantine (single or mixed) |
|  |  | Material of quarantine perimeter fencing |
|  |  | Is there double fencing around the quarantine area? |
|  |  | Dedicated boots for quarantine |
|  |  | Dedicated overalls for quarantine |
|  |  | Footbath with disinfectant solution for quarantine |
|  |  | Disposable protective equipment for quarantine staff (gloves + shoe covers + overalls) |
|  | Transport of incoming animals | Type of transport from off-site quarantine to replacement facility (own or hired) |
|  |  | Type of vehicle used for incoming replacements |
|  |  | Is the transport shared with other farms? |
|  |  | Shoe covers provided to drivers (off-site quarantine to farm trip) |
|  |  | Boots provided to drivers (off-site quarantine to farm trip) |
|  |  | Distance from off-site quarantine to the farm (km) |
|  |  | Type of transport from replacement facility to the farm (own or hired) |
|  |  | Type of vehicle used for transport to the farm |
|  |  | Is the transport to the farm shared with other farms? |
|  |  | Shoe covers provided to drivers (farm entry) |
|  |  | Boots provided to drivers (replacement to farm entry) |
|  |  | Distance from origin to farm (km) |
|  |  | Does the same journey bringing replacements also transport animals to the replacement unit? |
|  | Diagnostic testing for incoming animals | BVD test during quarantine |
|  |  | IBR test during quarantine |
|  |  | Neospora test during quarantine |
|  |  | Paratuberculosis test during quarantine |
|  |  | BVD test at replacement origin |
|  |  | IBR test at replacement origin |
|  |  | Neospora test at replacement origin |
|  |  | Paratuberculosis test at replacement origin |
| Farm access by visitors and vehicles | Farm perimeter fencing | Farm perimeter (m) |
|  |  | Perimeter fencing of the farm |
|  |  | Number of farm access points |
|  |  | Are access points controlled? |
|  |  | Number of footbaths |
|  |  | Type of fencing (single or mixed) |
|  |  | Material of perimeter fencing |
|  |  | Is there double fencing around the farm perimeter? |
|  |  | Lifting barrier |
|  |  | Number of lifting barriers |
|  |  | Manual swing gate |
|  |  | Number of manual swing gates |
|  |  | Automatic swing gate |
|  |  | Number of automatic swing gates |
|  |  | Manual sliding gate |
|  |  | Number of manual sliding gates |
|  |  | Automatic sliding gate |
|  |  | Number of automatic sliding gates |
|  |  | Disinfection arch |
|  | Visitor control | Number of different visitors in contact with animals per year |
|  |  | Total frequency of visits involving animal contact per month (excluding drivers) |
|  |  | Farm-provided boots for regular visitors (in contact with animals) |
|  |  | Farm-provided overalls for regular visitors (in contact with animals) |
|  |  | Footbath with disinfectant solution (for visitors only) |
|  |  | Disposable protective equipment for visitors (gloves + shoe covers + overalls) |
|  | Transport of outgoing animals | Frequency of vehicle entries (per month) |
|  |  | Shoe covers provided to drivers (outgoing trips) |
|  |  | Boots provided to drivers |
|  |  | Type of transport to slaughterhouse (own or hired) |
|  |  | Type of vehicle for slaughterhouse trips |
|  |  | Is transport shared with other farms? |
|  |  | Number of animal shipments to slaughterhouse (annual) |
|  |  | Number of animals sent to slaughterhouse (annual) |
|  |  | Distance from farm to slaughterhouse (km) |
|  |  | Are animals separated before transport to slaughterhouse? |
|  |  | Does the driver come into contact with animals remaining on the farm? |
|  |  | Type of transport to fattening unit (own or hired) |
|  |  | Type of vehicle for fattening transport |
|  |  | Is transport shared with other farms? |
|  |  | Number of animal shipments to fattening (annual) |
|  |  | Number of animals sent to fattening (annual) |
|  |  | Distance from farm to fattening unit (km) |
|  |  | Are animals separated before transport to fattening? |
|  |  | Does the driver come into contact with animals remaining on the farm? |
|  |  | Type of transport to rearing unit (own or hired) |
|  |  | Type of vehicle for rearing transport |
|  |  | Is transport shared with other farms? |
|  |  | Number of animal shipments to rearing (annual) |
|  |  | Number of animals sent to rearing (annual) |
|  |  | Distance from farm to rearing unit (km) |
|  |  | Are animals separated before transport to rearing or slaughter? |
|  |  | Does the driver come into contact with animals remaining on the farm? |
|  | Mortality management | Paved area for mortality disposal |
|  |  | Fencing around mortality disposal area |
|  |  | Disposal service (annual fee) |
|  | Worker hygiene protocols | Farm-dedicated boots for workers |
|  |  | Overalls for workers |
|  |  | Disposable protective equipment for workers (gloves + shoe covers + overalls) |
| Feed storage and water treatment | Feed storage | Number of 7,000 kg silos |
|  |  | Number of 10,000 kg silos |
|  |  | Number of 17,000 kg silos |
|  |  | Lidded feed bins |
|  |  | Number of lidded feed bins |
|  |  | Pallets used to keep buckets off the ground |
|  |  | Number of pallets |
|  | Drinking water treatment | Chlorine water treatment |
|  |  | Hydrogen peroxide water treatment |
| Pest control | Rodent control | Rodent control measures |
|  |  | Number of physical rodent traps |
|  |  | Number of bait stations |
|  |  | Cost of rodent control only |
|  | Flying and crawling insect control | Control of flying/biting insects |
|  |  | Control of crawling insects/cockroaches |
|  |  | Number of traps for flying/biting insects |
|  |  | Chemical control of flying/biting insects |
|  |  | Annual cost of chemical control of flying/biting insects |
|  |  | Monthly time dedicated to flying insect control (hours) |
|  |  | Number of cockroach traps |
|  |  | Cost of fly control only |
|  |  | Cost of cockroach control only |

**Table 3.S1.** **Production and health parameters of the three farms used in SimHerd to estimate the cost of disease introduction.**

| Parameter | Unit of measurement | Farm A | Farm B | Farm C |
| --- | --- | --- | --- | --- |
| Cow-years | Number | 724 | 247 | 79 |
| Liter milk per cow-year | Liter | 13,141 | 13,602 | 9,567 |
| Percent milk delivered | % | 98.9 | 98.9 | 93.0 |
| Bulk Tank SCC (x1000) | x 1000 cells/ml | 281 | 148 | 180 |
| Number of calvings | Number | 646 | 214 | 60 |
| Number of first parity calvings | Number | 167 | 104 | 35 |
| Stillbirth | % | 5.0 | 0.8 | 2.0 |
| Calf mortality, after birth | % | 8.0 | 0.4 | 2.0 |
| Cow mortality | % | 5 | 11 | 9 |
| Dystocia | Cases/100 cow-years | 2 | 2 | 2 |
| Milk Fever | Cases/100 cow-years | 6 | 6 | 6 |
| Retained Placenta | Cases/100 cow-years | 8 | 8 | 8 |
| Metritis | Cases/100 cow-years | 5 | 5 | 5 |
| Start breeding, first parity cows | Days after calving | 42 | 42 | 42 |
| Ketosis | Cases / 100 cow-years | 6 | 6 | 6 |
| Displaced Abomasum | Cases / 100 cow-years | 1 | 1 | 1 |
| Mastitis | Cases / 100 cow-years | 60 | 20 | 26 |
| Digital Dermatitis | Cases / 100 cow-years | 25 | 25 | 25 |
| Foul in the Foot | Cases / 100 cow-years | 4 | 4 | 4 |
| Claw and Leg Problems | Cases / 100 cow-years | 25 | 25 | 25 |
| Age at first calving | Months | 25 | 25 | 25 |
| Start breeding, heifers | Months | 13.9 | 13.3 | 15.8 |
| Heat observation rate, heifers | % | 55 | 64 | 37 |
| Conception rate, heifers | % | 41 | 43 | 24 |
| Start breeding | Days | 70 | 70 | 70 |
| Heat observation rate, cows | % | 63.0 | 65.0 | 40.3 |
| Conception rate, cows | % | 36 | 49 | 31 |
| Replacement rate | % | 27 | 30 | 38 |
| Yield first parity cows / older cows | % | 80 | 89 | 89 |

**Annex 2. Outputs obtained from the SimHerd simulation.**

**Table 1.S2. Contribution margin across the first 5 years of simulation for Farm A. The table presents the gross margin (GM) obtained from the simulation using the farm’s original parameters (standard), as well as the GM under the scenario in which the farm's productivity parameters were modified (scenario).**

| Year of simulation | GM Standard | GM Scenario |
| --- | --- | --- |
| Year 1 | 2,558,391 € | 2,404,019 € |
| Year 2 | 2,537,029 € | 2,360,193 € |
| Year 3 | 2,567,494 € | 2,417,324 € |
| Year 4 | 2,561,577 € | 2,416,841 € |
| Year 5 | 2,558,255 € | 2,415,014 € |

**Table 2.S2. Contribution margin across the first 5 years of simulation for Farm B. The table presents the gross margin (GM) obtained from the simulation using the farm’s original parameters (standard), as well as the GM under the scenario in which the farm's productivity parameters were modified (scenario).**

| Year of simulation | GM Standard | GM Scenario |
| --- | --- | --- |
| Year 1 | 804,074 € | 754,233 € |
| Year 2 | 811,393 € | 764,063 € |
| Year 3 | 808,975 € | 758,756 € |
| Year 4 | 804,473 € | 763,533 € |
| Year 5 | 804,251 € | 760,650 € |

**Table 3.S2. Contribution margin across the first 5 years of simulation for Farm C. The table presents the gross margin (GM) obtained from the simulation using the farm’s original parameters (standard), as well as the GM under the scenario in which the farm's productivity parameters were modified (scenario).**

| Year of simulation | GM Standard | GM Scenario |
| --- | --- | --- |
| Year 1 | 157,259 € | 156,868 € |
| Year 2 | 150,172 € | 135,197 € |
| Year 3 | 152,593 € | 140,590 € |
| Year 4 | 151,172 € | 142,852 € |
| Year 5 | 150,096 € | 140,901 € |

**Table 4.S2. Selected parameter values resulting from the SimHerd simulation for the study farms. The table presents a selection of outputs (not all parameters simulated by SimHerd), showing the average values over five simulation years for both the “Standard” scenario (using the farm’s original parameters) and the “Scenario” with modified productivity parameters.**

| Parameter of the farm | Farm A | | Farm B | | Farm C | |
| --- | --- | --- | --- | --- | --- | --- |
|  | **Standard** | **Scenario** | **Standard** | **Scenario** | **Standard** | **Scenario** |
| Milk yield per cow-year, kg ECM | 13,232 | 12,998 | 13,287 | 13,056 | 8,685 | 8,565 |
| Feed intake in FE per cow-year | 8,262 | 8,150 | 8,299 | 8,192 | 6,170 | 6,103 |
| Number of cow-years | 725 | 724 | 234 | 234 | 72 | 71 |
| Number of calvings | 756 | 739 | 255 | 251 | 66 | 60 |
| Replacement rate | 30.5 | 36 | 30.6 | 36.1 | 42.8 | 45.8 |
| Inseminations per cow-year (# on cows + heifers) | 3.5 | 4.3 | 3.1 | 3.9 | 3.4 | 3.9 |
| Number of first parity cows | 226 | 272 | 70 | 84 | 36 | 41 |
| Number of second parity cows | 167 | 182 | 53 | 58 | 19 | 18 |
| Number of third and older parity cows | 332 | 270 | 112 | 93 | 17 | 12 |
| Revenues milk | 4,512,975 € | 4,431,685 € | 1,480,622 € | 1,454,332 € | 296,753 € | 287,531 € |
| Total revenues | 4,873,978 € | 4,725,906 € | 1,616,598 € | 1,571,222 € | 356,356 € | 351,227 € |
| Expenses Feed, cows | 1,672,104 € | 1,649,304 € | 592,527 € | 584,789 € | 129,015 € | 125,388 € |
| Total expenses | 2,317,438 € | 2,313,478 € | 812,464 € | 810,580 € | 206,892 € | 210,278 € |

**Annex 3. Decision analysis on the most cost-effective biosecurity improvements.**

**Table 1.S3. Results from the decision analysis for Farm A.**

| Scenarios |  | Probability of disease introduction | | | | | | | | | |
| --- | --- | --- | --- | --- | --- | --- | --- | --- | --- | --- | --- |
|  |  | **Median value (50th percentile)** | | | **Minimum value (2,5% percentile)** | | | **Maximum value (97,5% percentile)** | | | |
|  |  | **Disease** | **No Disease** | **EMV^a^** | **Disease** | **No Disease** | **EMV^a^** | **Disease** | **No Disease** | **EMV^a^** |  |
| Current level | **Euro€** | -173,840 € | -19,969 € | -24,064 € | -173,840 € | -19,969 € | -21,489 € | -173,840 € | -19,969 € | -28,693 € |  |
|  | **p** | 2**.**66**%** | 97.34% |  | 0.99% | 99.01% |  | 5.67% | 94.33% |  |  |
|  | **Euro€*p** | -4,627 € | -19,437 € |  | -1,718 € | -19,771 € |  | -9,857 € | -18,836 € |  |  |
| Provide boots to all drivers | **Euro€** | -173,864 € | -19,993 € | -22,384 € | -173,864 € | -19,993 € | -20,881 € | -173,864 € | -19,993 € | -25,086 € |  |
|  | **p** | 1.55% | 98.45% |  | 0.58% | 99.42% |  | 3.31% | 96.69% |  |  |
|  | **Euro€*p** | -2,702 € | -19,683 € |  | -1,003 € | -19,878 € |  | -5,755 € | -19,331 € |  |  |
| Do not share equipment with other farms | **Euro€** | -173,866 € | -19,995 € | -23,902 € | -173,866 € | -19,995 € | -21,445 € | -173,866 € | -19,995 € | -28,318 € |  |
|  | **p** | 2.54% | 97.46% |  | 0.94% | 99.06% |  | 5.41% | 94.59% |  |  |
|  | **Euro€*p** | -4,415 € | -19,487 € |  | -1,639 € | -19,806 € |  | -9,404 € | -18,913 € |  |  |
| Provide boots to all visitors | **Euro€** | -173,864 € | -19,993 € | -23,912 € | -173,864 € | -19,993 € | -21,448 € | -173,864 € | -19,993 € | -28,342 € |  |
|  | **p** | 2.55% | 97.45% |  | 0.95% | 99.05% |  | 5.43% | 94.57% |  |  |
|  | **Euro€*p** | -4,428 € | -19,484 € |  | -1,644 € | -19,804 € |  | -9,433 € | -18,908 € |  |  |

**^a^**EMV= Expected monetary value.

**Table 2.S3. Results from the decision analysis for Farm B.**

| Scenarios |  | Probability of disease introduction | | | | | | | | | |
| --- | --- | --- | --- | --- | --- | --- | --- | --- | --- | --- | --- |
|  |  | **Median value (50th percentile)** | | | **Minimum value (2,5% percentile)** | | | **Maximum value (97,5% percentile)** | | | |
|  |  | **Disease** | **No Disease** | **EMV^a^** | **Disease** | **No Disease** | **EMV^a^** | **Disease** | **No Disease** | **EMV^a^** |  |
| Current level | **Euro€** | -57,401 € | -11,015 € | -18,297 € | -57,401 € | -11,015 € | -15,700 € | -57,401 € | -11,015 € | -20,988 € |  |
|  | **p** | 15.70% | 84.30% |  | 10.10% | 89.90% |  | 21.50% | 78.50% |  |  |
|  | **Euro€*p** | -9,012 € | -9,286 € |  | -5,798 € | -9,902 € |  | -12,341 € | -8,647 € |  |  |
| Provide boots to all drivers | **Euro€** | -62,599 € | -16,213 € | -17,917 € | -62,599 € | -16,213 € | -17,309 € | -62,599 € | -16,213 € | -18,546 € |  |
|  | **p** | 3.67% | 96.33% |  | 2.36% | 97.64% |  | 5.03% | 94.97% |  |  |
|  | **Euro€*p** | -2,299 € | -15,617 € |  | -1,479 € | -15,830 € |  | -3,149 € | -15,397 € |  |  |
| Do not share equipment with other farms | **Euro€** | -57,426 € | -11,039 € | -17,721 € | -57,426 € | -11,039 € | -15,338 € | -57,426 € | -11,039 € | -20,189 € |  |
|  | **p** | 14.40% | 85.60% |  | 9.27% | 90.73% |  | 19.73% | 80.27% |  |  |
|  | **Euro€*p** | -8,272 € | -9,449 € |  | -5,321 € | -10,017 € |  | -11,328 € | -8,862 € |  |  |
| Provide boots to all visitors | **Euro€** | -57,427 € | -11,041 € | -18,264 € | -57,427 € | -11,041 € | -15,688 € | -57,427 € | -11,041 € | -20,933 € |  |
|  | **p** | 15.57% | 84.43% |  | 10.02% | 89.98% |  | 21.32% | 78.68% |  |  |
|  | **Euro€*p** | -8,942 € | -9,322 € |  | -5,753 € | -9,935 € |  | -12,246 € | -8,687 € |  |  |

**^a^**EMV= Expected monetary value.

**Table 3.S3. Results from the decision analysis for Farm C.**

| Scenarios |  | Probability of disease introduction | | | | | | | | | |
| --- | --- | --- | --- | --- | --- | --- | --- | --- | --- | --- | --- |
|  |  | **Median value (50th percentile)** | | | **Minimum value (2,5% percentile)** | | | **Maximum value (97,5% percentile)** | | | |
|  |  | **Disease** | **No Disease** | **EMV^a^** | **Disease** | **No Disease** | **EMV^a^** | **Disease** | **No Disease** | **EMV^a^** |  |
| Current level | **Euro€** | -14,674 € | -5,697 € | -5,729 € | -14,674 € | -5,697 € | -5,710 € | -14,674 € | -5,697 € | -5,762 € |  |
|  | **p** | 0.36% | 99.64% |  | 0.15% | 99.85% |  | 0.73% | 99.27% |  |  |
|  | **Euro€*p** | -53 € | -5,676 € |  | -22 € | -5,688 € |  | -107 € | -5,655 € |  |  |
| Provide boots to all drivers | **Euro€** | -14,686 € | -5,709 € | -5,729 € | -14,686 € | -5,709 € | -5,717 € | -14,686 € | -5,709 € | -5,750 € |  |
|  | **p** | 0.22% | 99.78% |  | 0.09% | 99.91% |  | 0.45% | 99.55% |  |  |
|  | **Euro€*p** | -33 € | -5,696 € |  | -14 € | -5,704 € |  | -66 € | -5,683 € |  |  |
| Do not share equipment with other farms | **Euro€** | -14,698 € | -5,721 € | -5,751 € | -14,698 € | -5,721 € | -5,734 € | -14,698 € | -5,721 € | -5,781 € |  |
|  | **p** | 0.33% | 99.67% |  | 0.14% | 99.86% |  | 0.67% | 99.33% |  |  |
|  | **Euro€*p** | -48 € | -5,703 € |  | -20 € | -5,714 € |  | -98 € | -5,683 € |  |  |
| Provide boots to all visitors | **Euro€** | -14,700 € | -5,723 € | -5,753 € | -14,700 € | -5,723 € | -5,736 € | -14,700 € | -5,723 € | -5,783 € |  |
|  | **p** | 0.33% | 99.67% |  | 0.14% | 99.86% |  | 0.67% | 99.33% |  |  |
|  | **Euro€*p** | -49 € | -5,704 € |  | -20 € | -5,715 € |  | -98 € | -5,685 € |  |  |

**^a^**EMV= Expected monetary value.

**Table 4.S3. Sensitivity analysis of the probability of Bovine Viral Diarrhoea (BVD) introduction on the outcomes of the decision analysis.**

| Probability of introduction (Pintro) | EMV ^a^ of current level | Provide boots to all drivers | | | Do not share equipment with other farms | | | Provide boots to all visitors | | |
| --- | --- | --- | --- | --- | --- | --- | --- | --- | --- | --- |
|  |  | **Reduced Pintro** | **EMV ^a^ (€)** | **Relative change in EMV ^a^ scenario vs current level (%)** | **Reduced Pintro** | **EMV ^a^ (€)** | **Relative change in EMV ^a^ scenario vs current level (%)** | **Reduced Pintro** | **EMV ^a^ (€)** | **Relative change in EMV ^a^ scenario vs current level (%)** |
| 2% | - 23,046.01 € | 1,2% | - 21,789.76 € | 5.5% | 1,9% | -22,930.64 € | 0.5% | 1,9% | - 22,938.09 € | 0.5% |
| 4% | - 26,123.43 € | 2,3% | - 23,586.30 € | 9.7% | 3,8% | -25,866.36 € | 1.0% | 3,8% | - 25,882.96 € | 0.9% |
| 6% | - 29,200.85 € | 3,5% | - 25,382.83 € | 13.1% | 5,7% | -28,802.07 € | 1.4% | 5,7% | - 28,827.82 € | 1.3% |
| 8% | - 32,278.27 € | 4,7% | - 27,179.36 € | 15.8% | 7,6% | -31,737.79 € | 1.7% | 7,7% | - 31,772.68 € | 1.6% |
| 10% | - 35,355.69 € | 5,8% | - 28,975.90 € | 18.0% | 9,5% | -34,673.50 € | 1.9% | 9,6% | - 34,717.55 € | 1.8% |
| 12% | - 38,433.11 € | 7,0% | - 30,772.43 € | 19.9% | 11,4% | -37,609.21 € | 2.1% | 11,5% | - 37,662.41 € | 2.0% |
| 14% | - 41,510.53 € | 8,2% | - 32,568.96 € | 21.5% | 13,4% | -40,544.93 € | 2.3% | 13,4% | - 40,607.27 € | 2.2% |
| 16% | - 44,587.95 € | 9,3% | - 34,365.49 € | 22.9% | 15,3% | -43,480.64 € | 2.5% | 15,3% | - 43,552.14 € | 2.3% |
| 18% | - 47,665.37 € | 10,5% | - 36,162.03 € | 24.1% | 17,2% | -46,416.35 € | 2.6% | 17,2% | - 46,497.00 € | 2.5% |
| 20% | - 50,742.79 € | 11,7% | - 37,958.56 € | 25.2% | 19,1% | -49,352.07 € | 2.7% | 19,1% | - 49,441.86 € | 2.6% |
| 22% | - 53,820.21 € | 12,8% | - 39,755.09 € | 26.1% | 21,0% | -52,287.78 € | 2.8% | 21,1% | - 52,386.73 € | 2.7% |
| 24% | - 56,897.63 € | 14,0% | - 41,551.63 € | 27.0% | 22,9% | -55,223.50 € | 2.9% | 23,0% | - 55,331.59 € | 2.8% |
| 26% | - 59,975.05 € | 15,2% | - 43,348.16 € | 27.7% | 24,8% | -58,159.21 € | 3.0% | 24,9% | - 58,276.45 € | 2.8% |
| 28% | - 63,052.47 € | 16,3% | - 45,144.69 € | 28.4% | 26,7% | -61,094.92 € | 3.1% | 26,8% | - 61,221.32 € | 2.9% |
| 30% | - 66,129.89 € | 17,5% | - 46,941.23 € | 29.0% | 28,6% | -64,030.64 € | 3.2% | 28,7% | - 64,166.18 € | 3.0% |
| 32% | - 69,207.31 € | 18,7% | - 48,737.76 € | 29.6% | 30,5% | -66,966.35 € | 3.2% | 30,6% | - 67,111.04 € | 3.0% |
| 34% | - 72,284.73 € | 19,8% | - 50,534.29 € | 30.1% | 32,4% | -69,902.07 € | 3.3% | 32,5% | - 70,055.91 € | 3.1% |
| 36% | - 75,362.15 € | 21,0% | - 52,330.82 € | 30.6% | 34,3% | -72,837.78 € | 3.3% | 34,4% | - 73,000.77 € | 3.1% |
| 38% | - 78,439.57 € | 22,2% | - 54,127.36 € | 31.0% | 36,3% | -75,773.49 € | 3.4% | 36,4% | - 75,945.63 € | 3.2% |
| 40% | - 81,516.99 € | 23,4% | - 55,923.89 € | 31.4% | 38,2% | -78,709.21 € | 3.4% | 38,3% | - 78,890.50 € | 3.2% |
| 42% | - 84,594.41 € | 24,5% | - 57,720.42 € | 31.8% | 40,1% | -81,644.92 € | 3.5% | 40,2% | - 81,835.36 € | 3.3% |
| 44% | - 87,671.83 € | 25,7% | - 59,516.96 € | 32.1% | 42,0% | -84,580.63 € | 3.5% | 42,1% | - 84,780.23 € | 3.3% |
| 46% | - 90,749.25 € | 26,9% | - 61,313.49 € | 32.4% | 43,9% | -87,516.35 € | 3.6% | 44,0% | - 87,725.09 € | 3.3% |
| 48% | - 93,826.67 € | 28,0% | - 63,110.02 € | 32.7% | 45,8% | -90,452.06 € | 3.6% | 45,9% | - 90,669.95 € | 3.4% |
| 50% | - 96,904.09 € | 29,2% | - 64,906.56 € | 33.0% | 47,7% | -93,387.78 € | 3.6% | 47,8% | - 93,614.82 € | 3.4% |

^a^ Expected monetary value.

**Table 5.S3. Sensitivity analysis of the cost of a Bovine Viral Diarrhoea (BVD) outbreak on the outcomes of the decision analysis.**

| BVD outbreak cost | EMV ^a^ of current level | Provide boots to all drivers | | Do not share equipment with other farms | | Provide boots to all visitors | |
| --- | --- | --- | --- | --- | --- | --- | --- |
|  |  | **EMV ^a^ (€)** | **Relative change in EMV ^a^ scenario vs current level (%)** | **EMV ^a^ (€)** | **Relative change in EMV scenario vs current level (%)** | **EMV ^a^ (€)** | **Relative change in EMV ^a^ scenario vs current level (%)** |
| -153,871.00 € | - 24,064.11 € | - 22,384.11 € | 7.0% | -23,901.87 € | 0.7% | - 23,912.34 € | 0.6% |
| -169,258.10 € | - 24,473.66 € | - 22,623.20 € | 7.6% | -24,292.56 € | 0.7% | - 24,304.25 € | 0.7% |
| -184,645.20 € | - 24,883.22 € | - 22,862.29 € | 8.1% | -24,683.25 € | 0.8% | - 24,696.16 € | 0.8% |
| -200,032.30 € | - 25,292.77 € | - 23,101.37 € | 8.7% | -25,073.95 € | 0.9% | - 25,088.08 € | 0.8% |
| -215,419.40 € | - 25,702.32 € | - 23,340.46 € | 9.2% | -25,464.64 € | 0.9% | - 25,479.99 € | 0.9% |
| -230,806.50 € | - 26,111.87 € | - 23,579.55 € | 9.7% | -25,855.33 € | 1.0% | - 25,871.90 € | 0.9% |
| -246,193.60 € | - 26,521.43 € | - 23,818.64 € | 10.2% | -26,246.03 € | 1.0% | - 26,263.81 € | 1.0% |
| -261,580.70 € | - 26,930.98 € | - 24,057.73 € | 10.7% | -26,636.72 € | 1.1% | - 26,655.72 € | 1.0% |
| -276,967.80 € | - 27,340.53 € | - 24,296.81 € | 11.1% | -27,027.41 € | 1.1% | - 27,047.63 € | 1.1% |
| -292,354.90 € | - 27,750.08 € | - 24,535.90 € | 11.6% | -27,418.11 € | 1.2% | - 27,439.54 € | 1.1% |
| -307,742.00 € | - 28,159.64 € | - 24,774.99 € | 12.0% | -27,808.80 € | 1.2% | - 27,831.45 € | 1.2% |

^a^ Expected monetary value.

**Table 6.S3. Sensitivity analysis of biosecurity costs on the outcomes of the decision analysis.**

| Biosecurity cost | EMV ^a^ of current level | Provide boots to all drivers | | Do not share equipment with other farms | | Provide boots to all visitors | |
| --- | --- | --- | --- | --- | --- | --- | --- |
|  |  | **EMV ^a^ (€)** | **Relative change in EMV ^a^ scenario vs current level (%)** | **EMV ^a^ (€)** | **Relative change in EMV scenario vs current level (%)** | **EMV ^a^ (€)** | **Relative change in EMV ^a^ scenario vs current level (%)** |
| -19,968.59 € | - 24,064.11 € | - 22,384.11 € | 7.0% | -23,901.87 € | 0.7% | - 23,912.34 € | 0.6% |
| -21,965.45 € | - 26,060.97 € | - 24,383.43 € | 6.4% | -25,901.36 € | 0.6% | - 25,911.67 € | 0.6% |
| -23,962.31 € | - 28,057.83 € | - 26,382.76 € | 6.0% | -27,900.85 € | 0.6% | - 27,910.99 € | 0.5% |
| -25,959.17 € | - 30,054.69 € | - 28,382.08 € | 5.6% | -29,900.34 € | 0.5% | - 29,910.31 € | 0.5% |
| -27,956.03 € | - 32,051.55 € | - 30,381.40 € | 5.2% | -31,899.84 € | 0.5% | - 31,909.63 € | 0.4% |
| -29,952.89 € | - 34,048.41 € | - 32,380.72 € | 4.9% | -33,899.33 € | 0.4% | - 33,908.96 € | 0.4% |
| -31,949.74 € | - 36,045.27 € | - 34,380.05 € | 4.6% | -35,898.82 € | 0.4% | - 35,908.28 € | 0.4% |
| -33,946.60 € | - 38,042.13 € | - 36,379.37 € | 4.4% | -37,898.32 € | 0.4% | - 37,907.60 € | 0.4% |
| -35,943.46 € | - 40,038.98 € | - 38,378.69 € | 4.1% | -39,897.81 € | 0.4% | - 39,906.93 € | 0.3% |
| -37,940.32 € | - 42,035.84 € | - 40,378.02 € | 3.9% | -41,897.30 € | 0.3% | - 41,906.25 € | 0.3% |
| -39,937.18 € | - 44,032.70 € | - 42,377.34 € | 3.8% | -43,896.80 € | 0.3% | - 43,905.57 € | 0.3% |

^a^ Expected monetary value.
